# Negative Reflecting Meta-Mirrors

**DOI:** 10.1038/s41598-017-06184-1

**Published:** 2017-07-18

**Authors:** Rui Yang, Dong Li, Dongxing Gao, Aofang Zhang, Bowei Hu, Pei Yang, Zhenya Lei, Jiacheng Li

**Affiliations:** School of Electronic Engineering, Xidian University, Xi’an, 710071 People’s Republic of China

## Abstract

Using the gradient phase discontinuities that meta-mirrors provide, we show that the incident wave can be reflected anomalously with a broad angle range of negative reflections. Such reversed behaviors promote the immediate applications for the planar meta-mirrors to steer the signals more arbitrarily and the convex meta-mirrors to focus and collimate electromagnetic fields. We practically implement these negative reflecting meta-mirrors through an arrangement of subwavelength ring patches and generate the desired phase distribution by also considering the incident angle. Finally, the experiments are carried out to verify the functionality of the convex meta-mirror firstly, and the performances of the planar meta-mirror are also tested by further building up a dual reflector system with the demonstration of obtaining the plane wave from the convex meta-mirror and then having the well collimated beam negative reflected by the planar meta-mirror. The proposed design should be readily applicable to a wide range of electromagnetic problems, especially for devising smart planar illusion devices, and highly directive antennas mounting on convex surfaces of various platforms.

## Introduction

As the counterpart notion of the negative refraction of electromagnetic fields^1, 2^, negative reflection refers to the phenomenon that the incident waves and the reflecting beams are also appearing in the same side of the normal. Such an inversed electromagnetic behavior has been numerically described at the planar interfaces associated with certain media that are owning strong chiral parameters, being uniaxial, or possessing the bi-anisotropic characteristics^3–5^. It has also been observed from the hybrid grounded metamaterial slab, photonic crystals, and the planar array of silver dimers, where the oblique incident signals can be backwards reflected and continue to travel in the counter-propagating direction^6–8^.

Very recently, the generalized Snell’s laws^9^ has been intensively studied and rapidly adopted in wide research areas, where we can simply employ different coating patterns of the meta-surfaces with manageable phases to control the propagation of the electromagnetic fields, thus initiate the quest for tangible applications that have established a fresh paradigm in the electromagnetic community^9–19^. Such a strategy also brings it to the fore when combined with meta-mirrors to manipulate the reflecting spectrums by properly devised coating surfaces on the ground plane and initiates several distinctive applications, such as analog computing^20^, programmable diffusion^21–25^, and skin cloaks^26^. Especially, it allows to redirect electromagnetic fields and reshape wave-fronts by simply arranging the coated phase distributions instead of constructing bulk dielectric layers with gradient refractive index or tailored geometry profile, thus offers very attractive solutions for building-up ultra-thin and conformal functional reflecting devices^27–31^. In this paper, we will show that the incident wave can be reflected anomalously with a broad angle range of negative reflections from the phase discontinuity meta-mirrors. Such reversed behaviors promote the immediate applications for the planar meta-mirrors to steer the signals more arbitrarily and the convex meta-mirrors to focus and collimate electromagnetic fields. We continue to practically implement these negative reflecting meta-mirrors through a periodic arrangement of the subwavelength ring patches, where the impacts of the oblique incident angles are also examined when generating the desired phase distributions. Finally, the experiments are carried out to verify the functionality of the convex meta-mirror firstly, and the performances of the planar meta-mirror are also tested by further building up a dual reflector system with the demonstration of obtaining the plane wave from the convex meta-mirror and then having the well collimated beam negative reflected by the planar meta-mirror. The proposed design should be readily applicable to a wide range of electromagnetic problems, especially for devising smart planar illusion devices, and highly directive antennas mounting on convex surfaces of various platforms.

**Figure 1:**
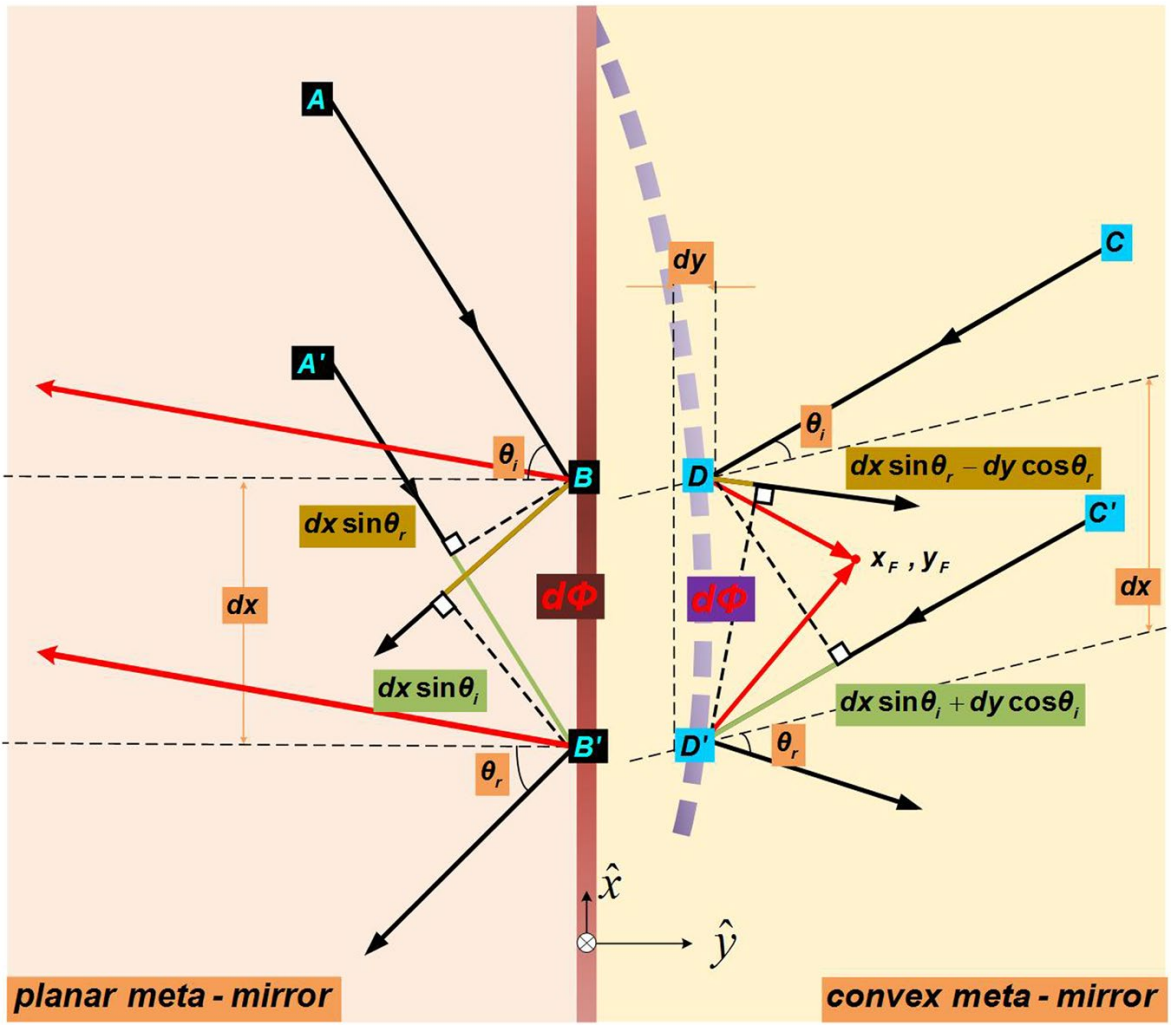
Design principle of the planar meta-mirror *BB*′ and the convex meta-mirror *DD*′. *B* and *B*′ on the planar meta-mirror are very closed to each other in positions with small variations of *dx*. *D* and *D*′ on the convex meta-mirror are very closed to each other in positions with small variations of *dx* and *dy*. *dΦ* refers to the phase variation between these two poisons of *B*(*D*) and *B*′(*D*′) on the meta-mirrors. The black rays refer the incidences and the normal behavior of the positive reflections. The red rays refer to the negative reflections.

Let us start with a planar meta-mirror under an oblique incident plane wave, as shown in Fig. 1. The relationship between the reflecting angle and the incident angle could be redefined according to the general Snell’s law by setting the phase distribution from the meta-mirror as $d{\rm{\Phi }}=k(\sin \,{\theta }_{r}-\,\sin \,{\theta }_{i})dx$, where *k* refers to the wavenumber in the free space, *θ*_*i*/*r*_ refers to the angles of the incidence and the reflection. Therefore, we can redirect the reflected beams to go in any directions even obtaining the negative *θ*_*r*_ as long as we properly tune the phase variation along the *x*-direction of the meta-mirror and make the wave path differences from the incidence $dx\,\sin \,{\theta }_{i}$ and the reflection *dx* sin *θ*_*r*_ always identical with each other to form the plane wave front. Based on the same principle, we can also redirect the reflecting beams from the convex meta-mirror as shown in Fig. 1, even to have the incident plane wave focused rather than diverged with the negative reflections. For instance, the desired reflection angle from the convex curvature (*x*, *y*) can be determined by ${\theta }_{r}(x,y)=\arctan [({x}_{F}-x)/({y}_{F}-y)]$ between a desired focal point of (*x*_*F*_, *y*_*F*_) and the well collimated radiations. Therefore, the reflection and the incidence can thus have the relationship of $d{\rm{\Phi }}=k[(\sin \,{\theta }_{r}-\,\sin \,{\theta }_{i})dx-(\cos \,{\theta }_{r}+\,\cos \,{\theta }_{i})dy]$, where the difference from the planar meta-mirror deign is that we have to include the impacts of phase variation in the *y*-direction from the convex meta-mirror.

**Figure 2:**
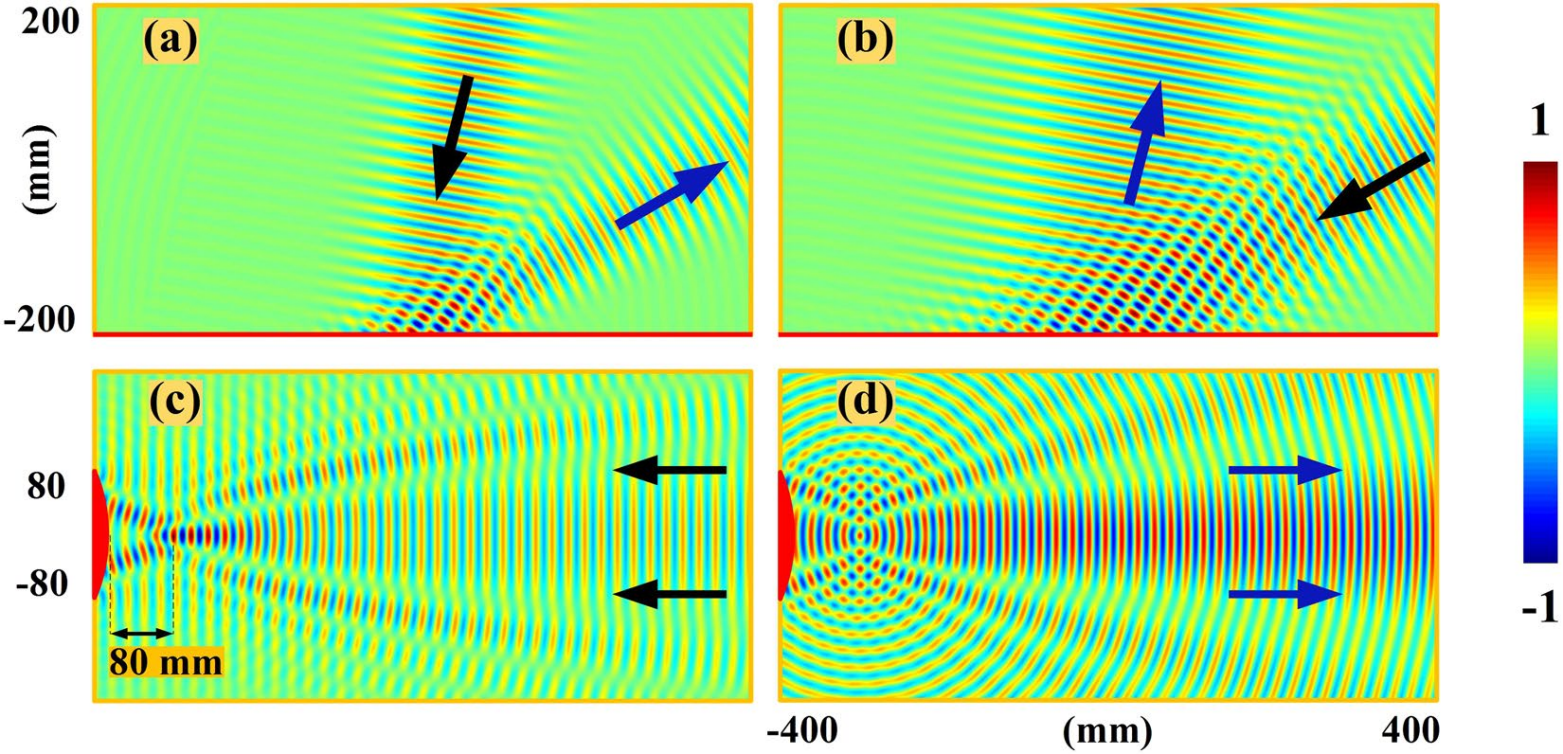
The near-fields of the negative reflection properties from the meta-mirrors. The demonstrations are based on the real part of the E-fields at 15 GHz, and the gradient phase layer of the meta-mirrors are all assumed to zero thickness using proper boundary conditions. (**a**) Planar meta-mirror with 10° incidence and −60° reflection. (**b**) Planar meta-mirror with 60° incidence and −10° reflection. (**c**) The focusing of rays from the convex meta-mirror when excited by a plane wave. (**d**) The collimation of rays from the convex meta-mirror when excited by a line source at the focal point.

To illustrate our design of the negative reflections from such meta-mirrors, we employ the method of moments program to perform the simulation where properly boundary conditions are used to define the phase distribution. To be more specifics, the surface current on the boundary are motivated by ${E}_{{\rm{in}}}{{\rm{e}}}^{j{{\rm{\Phi }}}_{0}}$ where *E*_in_ refers to the incident electromagnetic fields, and the Φ_0_ refers to the additional phases imposed to the original boundary of the perfect conductor. The scattering fields on the boundary can thus be defined as ${E}_{sc}=-{E}_{{\rm{in}}}{{\rm{e}}}^{j{{\rm{\Phi }}}_{0}}$ with the reflecting phase of ${\rm{\Phi }}={{\rm{\Phi }}}_{0}+\pi$. In this way, we can simply have the variations of reflecting phases on the boundary with the position information as $d{\rm{\Phi }}=k(\sin \,{\theta }_{r}-\,\sin \,{\theta }_{i})dx$ for the planar meta-surface to redirect the incidence and $d{\rm{\Phi }}=k[(\sin \,{\theta }_{r}-\,\sin \,{\theta }_{i})dx-(\cos \,{\theta }_{r}+\,\cos \,{\theta }_{i})dy]$ for the convex meta-surface to focus and collimate the electromagnetic fields. The gradient phase layer of meta-mirrors could be zero thickness but may still preserve the desired phase variation, so that we are able to exclude the multi-reflection effects caused by using the gradient dielectric media with finite thickness for the needed phase. Figure 2 thus demonstrates the near-fields of the negative reflection properties from the meta-mirrors at 15 GHz. With the setup of 6.5325 radians per wavelength for the phase variations on the planar meta-mirror, the oblique incidences of TE-polarized plane waves from 10° and 60° are reflected negatively in −60° and −10° respectively instead of going for the specular reflections, as shown in Fig. 2(a and b). On the other hand, we can also observe the focusing and collimation properties through the negative reflection from the well devised convex meta-mirrors at 15 GHz in Fig. 2(c and d), where the convex meta-mirror is chosen to be an eighth of a circle for simplicity with an aperture of *A* = 160 mm to have the preferred focal length of *f*_*L*_ = 80 mm. The phase variation can thus be set as ${\rm{\Phi }}(x)=k\sqrt{{x}^{2}-(r+{f}_{L}-\sqrt{{r}^{2}-{x}^{2}})}$, where *k* refers to the wave number in the free space and *r* = 210 mm refers to the radius of the convex curvature. As can be observed, the reflected beams from the convex meta-mirror is shown to be capable of focusing the incident plane wave in front the arc surface at the devised focal point, and the well collimated plane wave, on the other hand, can also be generated when we excite the convex mirror at the focal point of 80 mm in front of the convex meta-mirror.

**Figure 3:**
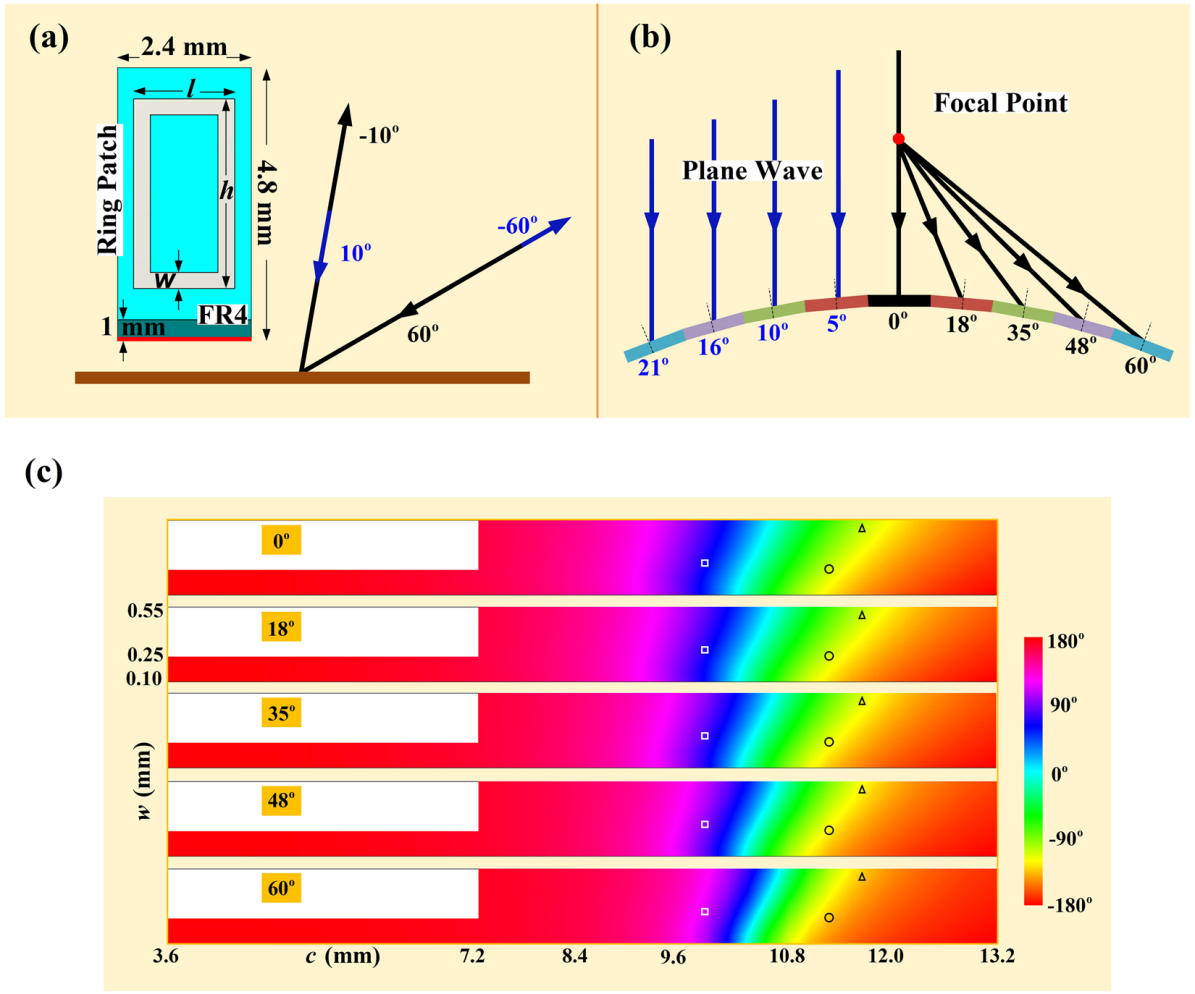
Practical implementation of the negative reflecting meta-mirrors using rectangular ring patches on the FR4 grounded slab. (**a**) Unit cell of the ring patch for the planar meta-mirror design. (**b**) Simplified structure with nine discrete polygonal surfaces for the convex meta-mirror with the structural merits of symmetry. (**c**) The relationships between the reflecting phases and the ring patches with different structural parameters when considering the incident angles. The square, ring and triangle marks refer to the ring patches having the size of (*c*, *w*) = (9.84 mm, 0.30 mm), (11.28 mm, 0.25 mm), and (11.76 mm, 0.50 mm).

We now consider the practical implementation of such negative reflecting meta-mirrors where the required phase distribution is obtained from the rectangular ring patches on the FR4 (*ε*_*r*_ = 4.4, loss tangent 0.02) grounded slab of 1 mm thick with the unit cell size of *P* = 2.4 × 4.8 mm^2^ as shown in Fig. 3(a). One group of 8 unit cells are set to have 360° phase variation with the gradient phase step of 45° for the case of 10° incidence and −60° reflection, or vice versa for the case of 60° incidence and −10° reflection from the planar meta-mirror to satisfy the initially devised phase distribution of 6.5325 radians per wavelength. The perimeter *c* = 2(*h* + *l*) and the width *w* of the ring patches are here functioning as the tunable parts to adjust the reflected phases, with the height *h* and the length *l* of the ring patches are always following the relationship of *h* = 2 *l* for simplicity. Therefore, different sizes of the ring patches should be employed to obtain the entire 360° phase variation for redirecting the reflections in the desire directions from the planar meta-mirror. However, we have to note that the responses of such ring patches are sensitive to the incidence angle, thus a tailor design of the ring patches should always include the influence of the incident angle. That is to say, the case of 60° incidence with −10° reflection and the case of 10° incidence with −60° reflection would require different ring patches, although the light paths between the incidence and the reflection are reversible. When comes to the convex meta-mirror as shown in Fig. 3(b), we employ nine discrete polygonal surfaces to reconstruct the convex curvature, where every polygonal surface has the length of 19.2 mm so as to hold eight ring patches with the same unit size of *P* = 2.4 × 4.8 mm^2^ as the case of planar meta-mirror. The total aperture of the convex meta-mirror thus has an aperture size of 168.03 mm with 9 groups of 8 unit cells. The oblique angles of the polygonal surfaces are set to be 0°, 5°, 10°, 16°, 21°, which are also be considered as the average incident angles on the polygonal surface with the illumination of a plane wave when the convex meta-mirror functions as a receiver. On the other hand, the average incident angles would thus be 0°, 18°, 35°, 48°, 60° when we feed it at the focal point by employing such a curvature as an emitting antenna. Due the sensitivity to the incident angle, every polygonal surface should have different arrangements of ring patches rather than the one periodic repeated on the planar meta-mirror. As a result, we would have 72 unit cells on the whole curvature of the convex meta-mirror with the structural merits of symmetry. Figure 3(c) then demonstrates the relationships between the reflecting phases and the ring patches with different structural parameters when considering the incident angles, where we only employ the convex mirror as the radiator to generate the well collimated beam and the planar meta-mirror are assumed to be solely under the 60° incidence of a plane wave in the following demonstration. We can observe that the reflecting phase would go increasing when the ring width becomes larger, and would be capable of experiencing the overall 360° phase variation when we enlarge the perimeter of the ring patch. We can also observe clearly that the reflecting phase varies as the incident angle changes, and the gradients of such difference turns more apparent especially when the incident angle becomes larger. The normal incidence and the 60° incidence have greatly difference reflecting phases with the same ring patches. For instance, the same ring patches having the dimensions of (*c*, *w*) = (9.84 mm, 0.30 mm), (11.28 mm, 0.25 mm), and (11.76 mm, 0.50 mm) would experience 58.52-, 21.43-, and 21.98-degree phase variations when under the normal incidence and the 60° oblique incidence.

**Figure 4:**
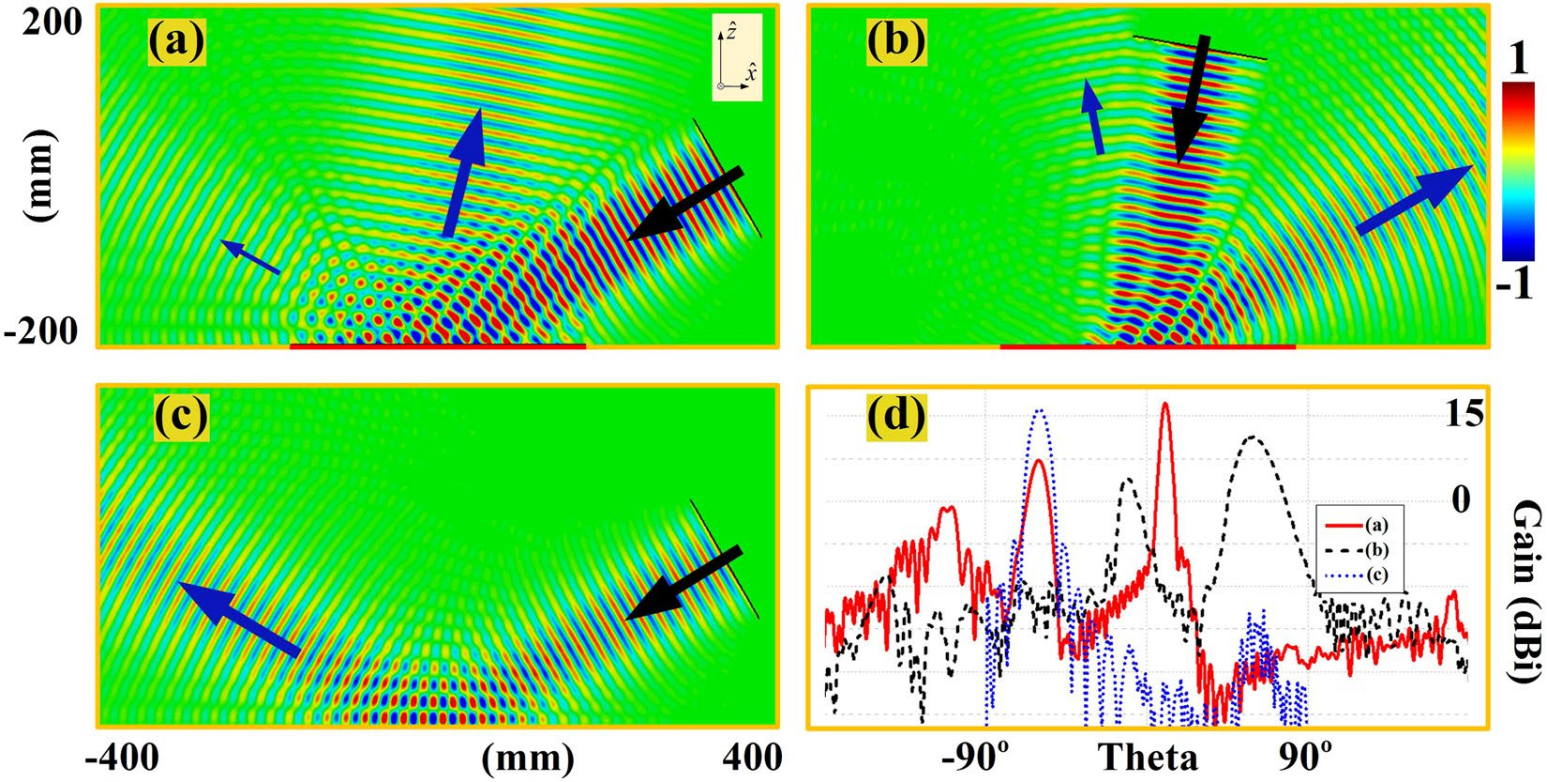
The radiation performances of the planar meta-mirror and conventional planar mirror. The meta-mirrors have the aperture of 345.6 mm with the incident plane wave from a 160 mm aperture wave port. (**a**) Planar meta-mirror with 60° incident plane wave. (**b**) Planar meta-mirror with 10° incident plane wave. (**c**) Conventional planar mirror with 60° incident plane wave. In (**a**,**b** and **c**), the real parts of E-fields are normalized by 550 V/m, 600 V/m, and 800 V/m respectively. (**d**) Far-field radiation patterns of the three cases in (**a**,**b** and **c**).

**Figure 5:**
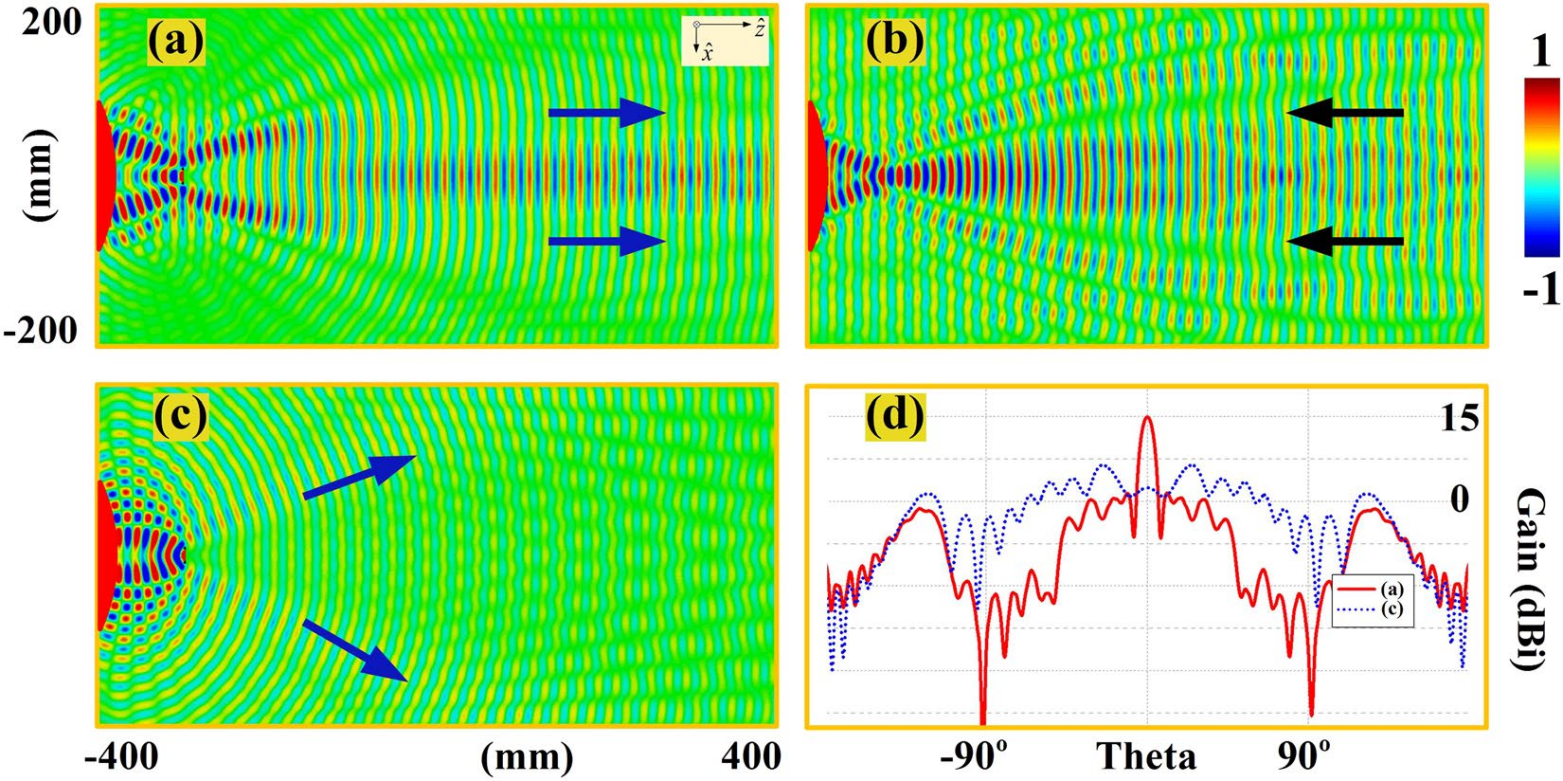
The radiation performances of the convex meta-mirror and conventional convex mirror. (**a**) The collimation of rays from the convex meta-mirror when excited by the WR62 waveguide at the focal point. (**b**) The focusing of rays from the convex meta-mirror when excited by a plane wave. (**c**) The divergence of rays from conventional convex mirror when excited by the WR62 waveguide. In (**a**,**b** and **c**), the real parts of E-fields are normalized by 550 V/m, 800 V/m, and 600 V/m respectively. (**d**) Far-field radiation patterns of the two cases in (**a** and **c**).

With these observations, we now employ different ring patches to build up the meta-mirrors for the devised functionalities, as shown in Tables 1 and 2. The 8 unit cells in 19.2 mm for the planar meta-mirror are set to have 360° phase variation with the gradient steps of 45° for the demonstration of the 60° incidence and −10° reflection. On the other hand, the other 5 groups of 8 unit cells for the convex meta-mirror design refer to the incident angle of 0°, 18°, 35°, 48°, 60° respectively with the excitation from the focal point to synthesize the directive beams, and the phase variation between two adjacent unit cell of the ring patches would not be uniform. Based on such a choice, we construct the planar meta-mirror and the convex meta-mirror, and perform the full wave simulations (CST microwave studio) in 10 mm high parallel plate waveguides having the dimension of 400 × 800 mm^2^ at 15 GHz. Figure 4 thus demonstrates the reflections from the same planar meta-mirror under a plane wave incidence in 60° and 10° respectively, and for the comparison purpose, we also present the conventional planar mirror without ring patches under a plane wave incidence from 60°. We can observe in Fig. 4(a) that the plane wave incidence from 60° is successfully reflected in the direction of −10° as we expected. However, the 10° incidence, as shown in Fig. 4(b), is not fully qualified to redirect coming signals into −60° with some part of the energy going for the specular reflection and the negative reflections are not well collimated into the plane wave for the same meta-mirror. This is because of the angular dispersion of the reflecting phase from the ring patches, and the meta-surface devised for the 60° incidence and −10° reflection is not fully qualified even for the reversible light path. We can also observe in Fig. 4(c) that the conventional mirror without ring patches under the 60° incidence only have the reflecting beams go into the 60° direction with normal behavior of the positive reflection. In the far-field plots as shown in Fig. 4(d), a distant observer also sees the directive beam with the negative reflection, and confirms our design with 17.44 dBi gain of the negative reflection and only 7.13 dBi of positive spectacular reflection when the meta-mirror is under the illumination of a 60° plane wave. However, the 10° incidence on the same meta-mirror has degraded radiations for the negative reflection with only 11.25 dBi gain. On the other hand, the conventional planar mirror only possesses the highly directive beam of positive spectacular reflection with the gain of 16.14 dBi. Figure 5 demonstrates the focusing and collimating properties of the convex meta-mirror, and also illustrates the performances of an ordinary convex mirror with the same structure. We can observe in Fig. 5(a) that a well collimated beam is generated from the convex meta-mirror when we feed it at the focal point by a WR62 waveguide. In the meanwhile, we can also observe that the convex meta-mirror still has the ability to focus large parts of the incident plane wave as shown in Fig. 5(b). This is because the average incidence difference of the convex meta-mirror is less than 40 degrees when it is functioning as a receiver and the emitting antennas, and such average incidence differences would not lead to huge variations in the reflecting phases as shown in Fig. 3(c), especially the average incidence difference of central part of the 5 polygonal surfaces is less than 25 degrees. On the other hand, we can observe in Fig. 5(c) that the conventional convex mirror without ring patches is having the reflecting beams diverged as the normal behavior of the positive reflection. Figure 5(d) thus gives the far-field plots of the convex meta-mirror as an emitting antenna, and the conventional convex mirror of the same structure without ring patches. We can observe that the well collimated beam is generated from our design and the directive beam has the gain of 15 dBi with the -3dB beamwidth of only 6.4 degrees. In the meanwhile, the conventional convex mirror only performs the scattering of the excitation with diverged radiations.

**Table 1:** The dimensions and the corresponding reflecting phases of the ring patches for the planar meta-mirror with 60° incidence and −10° reflection.

Planar Meta-mirror with 60° Incidence	*c* (mm)	9.00	9.84	10.08	10.32	10.80	10.80	11.40	13.20
	*w* (mm)	0.50	0.25	0.10	0.20	0.50	0.25	0.35	0.10
	reflecting phase (degrees)	135	90	45	0	−45	−90	−135	−180

**Table 2:** The dimensions and the corresponding reflecting phases of the ring patches for the convex meta-mirror with the excitation at the focal point.

ConvexMeta-mirror with 0° Incidence	*c* (mm)					13.20	13.20	13.20	13.20
	*w* (mm)					0.10	0.10	0.15	0.20
	reflecting phase (degrees)					−180	−179	−176	−172
Convex Meta-mirror with 18° Incidence	*c* (mm)	12.60	12.60	11.40	11.40	11.40	10.80	10.80	10.80
	*w* (mm)	0.25	0.50	0.15	0.30	0.50	0.25	0.40	0.55
	reflecting phase (degrees)	−163	−149	−133	−117	−99	−80	−61	−40
Convex Meta-mirror with 35° Incidence	*c* (mm)	10.20	10.20	9.84	9.60	8.64	3.60	13.20	12.00
	*w* (mm)	0.10	0.35	0.25	0.45	0.20	0.20	0.10	0.35
	reflecting phase (degrees)	−14	16	47	79	112	145	179	−146
Convex Meta-mirror with 48° Incidence	*c* (mm)	10.80	10.80	10.56	10.20	9.84	9.00	4.80	12.00
	*w* (mm)	0.15	0.40	0.40	0.30	0.25	0.15	0.20	0.25
	reflecting phase (degrees)	−107	−64	−21	22	66	111	156	−159
Convex Meta-mirror with 60° Incidence	*c* (mm)	10.80	10.80	10.32	10.08	9.84	4.80	12.00	10.80
	*w* (mm)	0.20	0.45	0.20	0.20	0.50	0.20	0.50	0.25
	reflecting phase (degrees)	−110	−58	−6	47	99	152	−155	−101

So far we have numerically tested all the designs of the negative reflection meta-mirrors, and the differences between the demonstrations in Figs 2, 4 and 5 are basically the ideal case simulations versus the ring patch based practical implementation. Therefore, we can estimate the efficiency of the proposed ring patch based meta-mirrors by compared these two simulations^16^. The meta-mirrors in Fig. 2 are assumed to be zero thickness with specific boundary condition to manipulate the wave-front and the direction of wave propagation, while the meta-mirrors in Figs 4(a) and 5(a) are based on ring patches of different sizes etched on the 1 mm thick FR4 substrate, and we have to discretize the originally continuous phase variation before the practical implementation. As a result, there should be some degradations in the radiation performances and the efficiency of the meta-mirrors in Figs 4(a) and 5(a) can thus be described by comparing the radiation performances with the ideal simulations in Fig. 2(b and d), as demonstrated in Table 3. Clearly, the employ of the ring patches to construct the proposed meta-mirrors would lead to degradations in the reflecting performances and thus turn out to have the lower efficiency. However, the overall performance of the meta-mirrors all fulfill the original targets of generating negative reflections.

**Table 3:** Comparisons of the ideal meta-mirrors in Fig. 2 and the ring patch based meta-mirrors in Figs 4 and 5.

Directivities of Ideal Meta-mirrors (dBi)	Gains of the Ring Patch based Meta-mirrors (dBi)	Efficiency (%)
19.05 in Fig. 2(b)	17.44 in Fig. 4(a)	69.02
16.17 in Fig. 2(d)	15.00 in Fig. 5(a)	76.38

**Figure 6:**
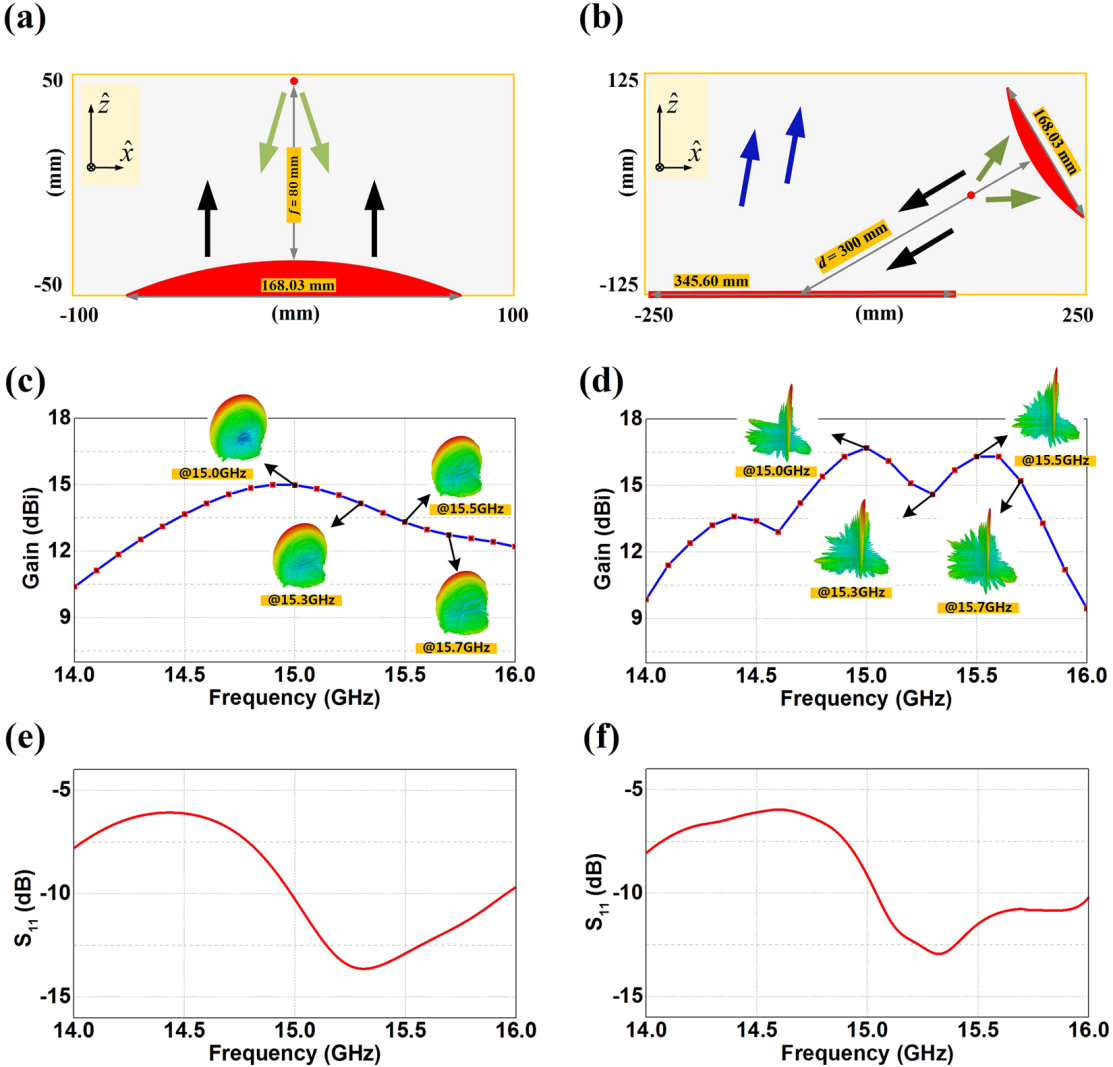
Configurations and the radiation performances of the optimized convex meta-mirror and the dual reflecting system. (**a**) The optimized convex meta-mirror. (**b**) The optimized dual reflecting system. (**c**) The gains of the optimized convex meta-mirror from 14 GHz to 16 GHz. (**d**) The gains of the optimized dual reflecting system from 14 GHz to 16 GHz. (**e**) The reflection coefficients of the optimized convex meta-mirror from 14 GHz to 16 GHz. (**f**) The reflection coefficients of the optimized dual reflecting system from 14 GHz to 16 GHz.

In order to experimentally test the proposed meta-mirrors, we optimize the dimensions of the parallel plate waveguide, and demonstrate how the size parameters would affect the reflecting performance. We start with the convex meta-mirror having the aperture of 168.03 mm, and finally find that the convex meta-mirror with 200 × 100 mm^2^ parallel plate waveguide would have almost the same radiation performance as the one with original 400 × 800 mm^2^ parallel plate cover, and the gains maintain around 15 dBi (see Supplementary Fig. S1(a and b)). For the verification of the planar meta-mirror, we propose the dual reflecting system by combining the convex meta-mirror and the planar meta-mirror. The convex meta-mirror is set to have the incident angle of 60° for the planar meta-mirror having an aperture size of 345.6 mm with 18 groups of 8 unit cells. We optimize the distance between the two meta-mirrors and the size of the parallel plate waveguide with the aim to achieve the satisfactory radiations and the smaller volume of the whole reflecting system, where we finally reach the gain of 16.69 dBi with 300 mm separation between the two meta-mirrors in the parallel plate waveguide having the dimension of 250 × 500 mm^2^ (see Supplementary Fig. S1(c and d)). Figure 6 thus demonstrate the final reached configurations of the optimized convex meta-mirror and the dual-reflecting system and their radiation performances from 14 GHz to 16 GHz. We can observe that the gains of both meta-mirrors vary fast as the frequency deviating from 15 GHz, and this should be reasonable because phase distributions on the meta-mirrors are very sensitive to the frequency change. The reflection coefficients from the feed rises sharply when the frequency decreases from 15 GHz of both demonstrations, and maintain above −10 dB. Therefore, the preferred working bandwidth of our designs would be from 15 GHz to 15.7 GHz. Within such a frequency range, the gains of the proposed convex meta-mirror are all above 12.73dBi with the peak value of 14.98 dBi at 15.0 GHz, and the reflection coefficient maintains around −10 dB varied from −10.27 dB to −13.63 dB. In the meanwhile, the gains of dual-reflecting meta-mirror system are all above 14.6 dBi with the peak value of 16.7 dBi at 15 GHz, and the reflection coefficient maintains around −10 dB varied from −9.18 dB to −12.94 dB.

**Figure 7:**
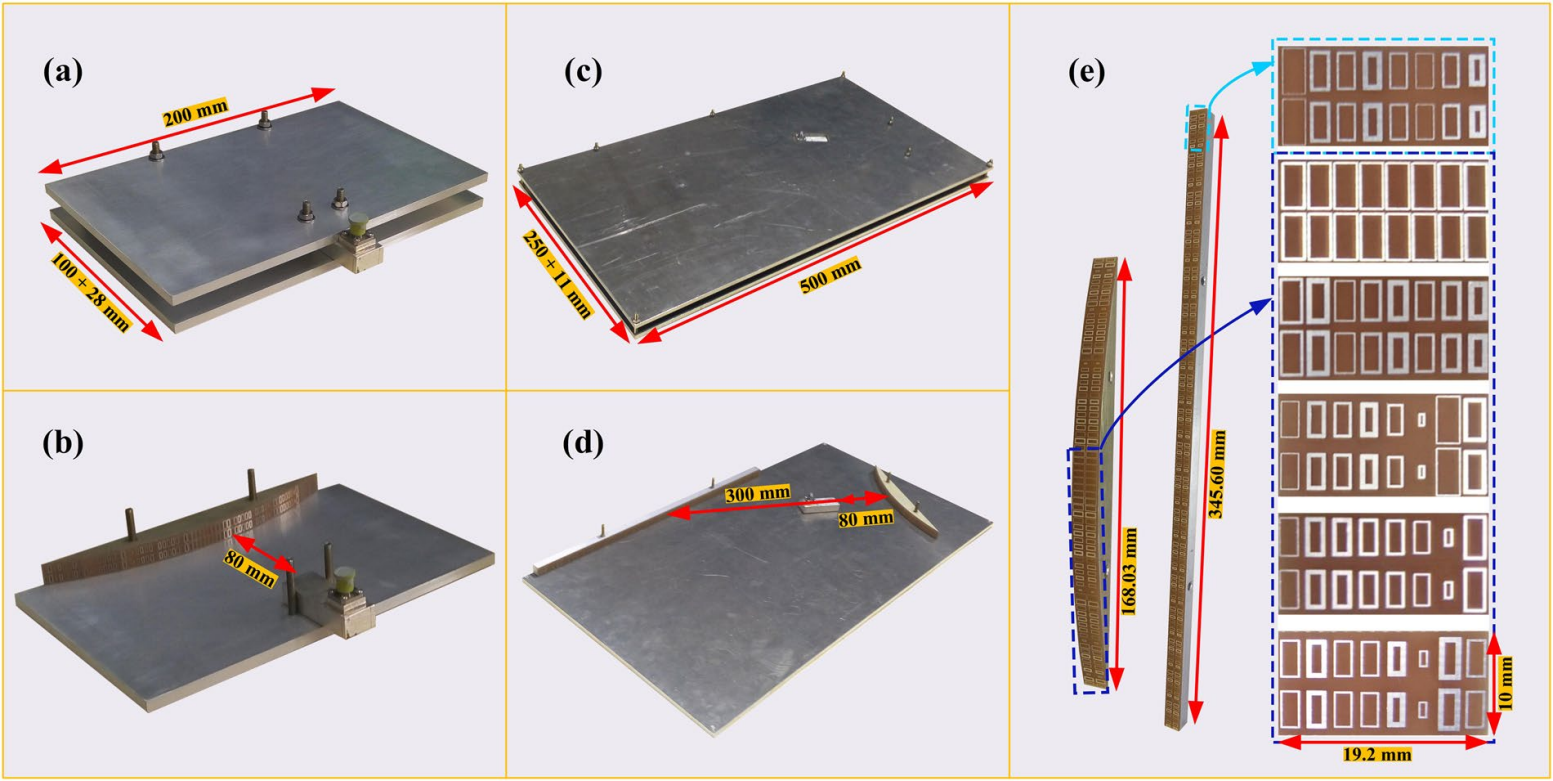
Manufactured photos of the optimized convex meta-mirror and the dual reflecting system. (**a**) The whole structure of the convex meta-mirror. The parallel plate waveguide has the size of (100 + 28) × 200 mm^2^, where the WR62 waveguide has additional 28 mm embedded length in the parallel plate waveguide in the experimental setup. (**b**) The whole convex meta-mirror without the upper plate. (**c**) The whole system of the dual reflecting system. The parallel plate waveguide has the size of (250 + 11) × 500 mm^2^, where the additional 11 mm is the total thickness of the supporting bracket and the planar meta-mirror. (**d**) The dual reflecting system without the upper plate. (**e**) Magnified pictures of the planar meta-mirror, the convex meta-mirror, and the ring patches.

**Figure 8:**
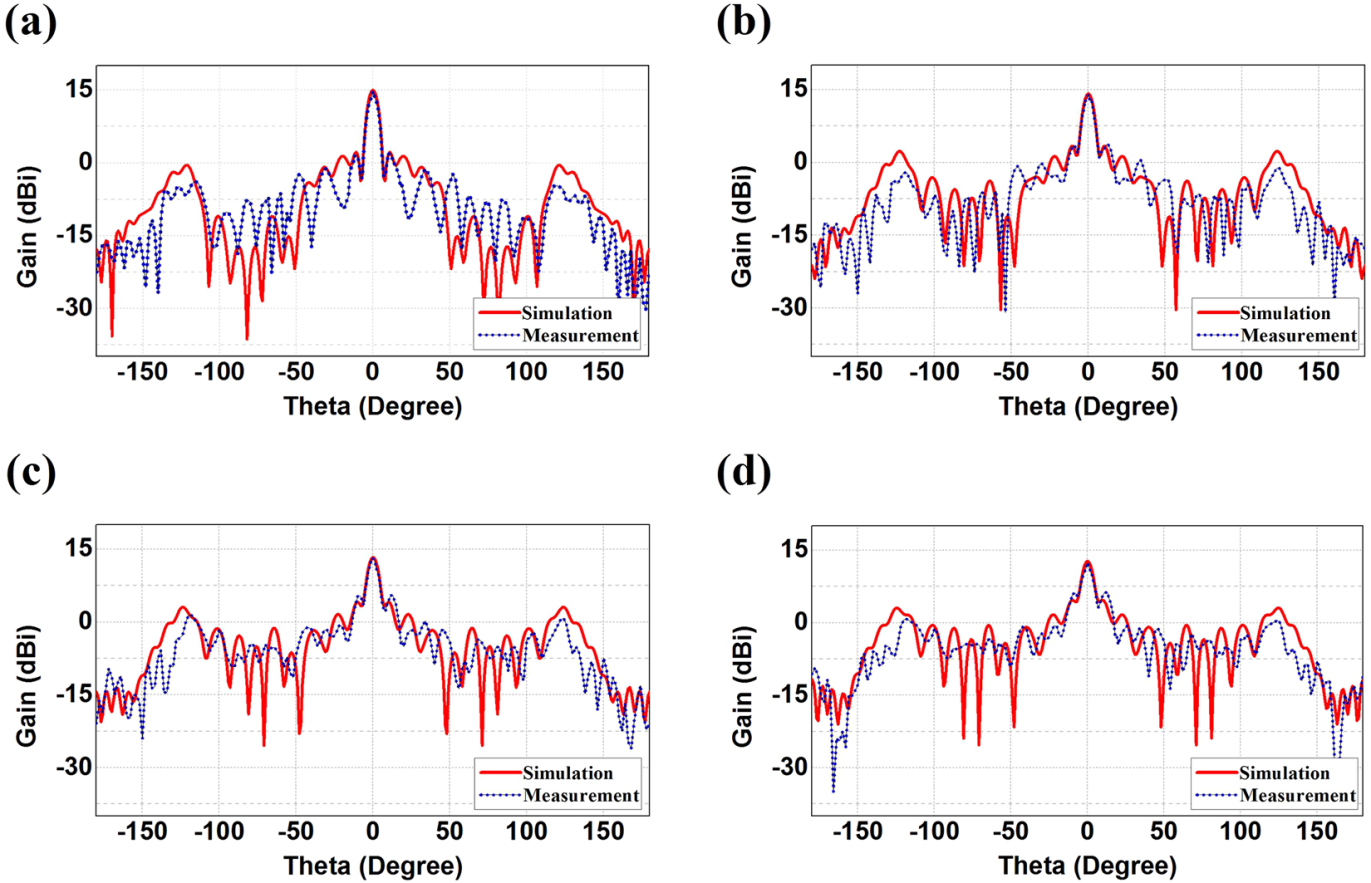
Comparisons of the measured radiation patterns and the simulation results of the convex meta-mirror. (**a**) 15 GHz, (**b**) 15.3 GHz, (**c**) 15.5 GHz, and (**d**) 15.7 GHz.

**Figure 9:**
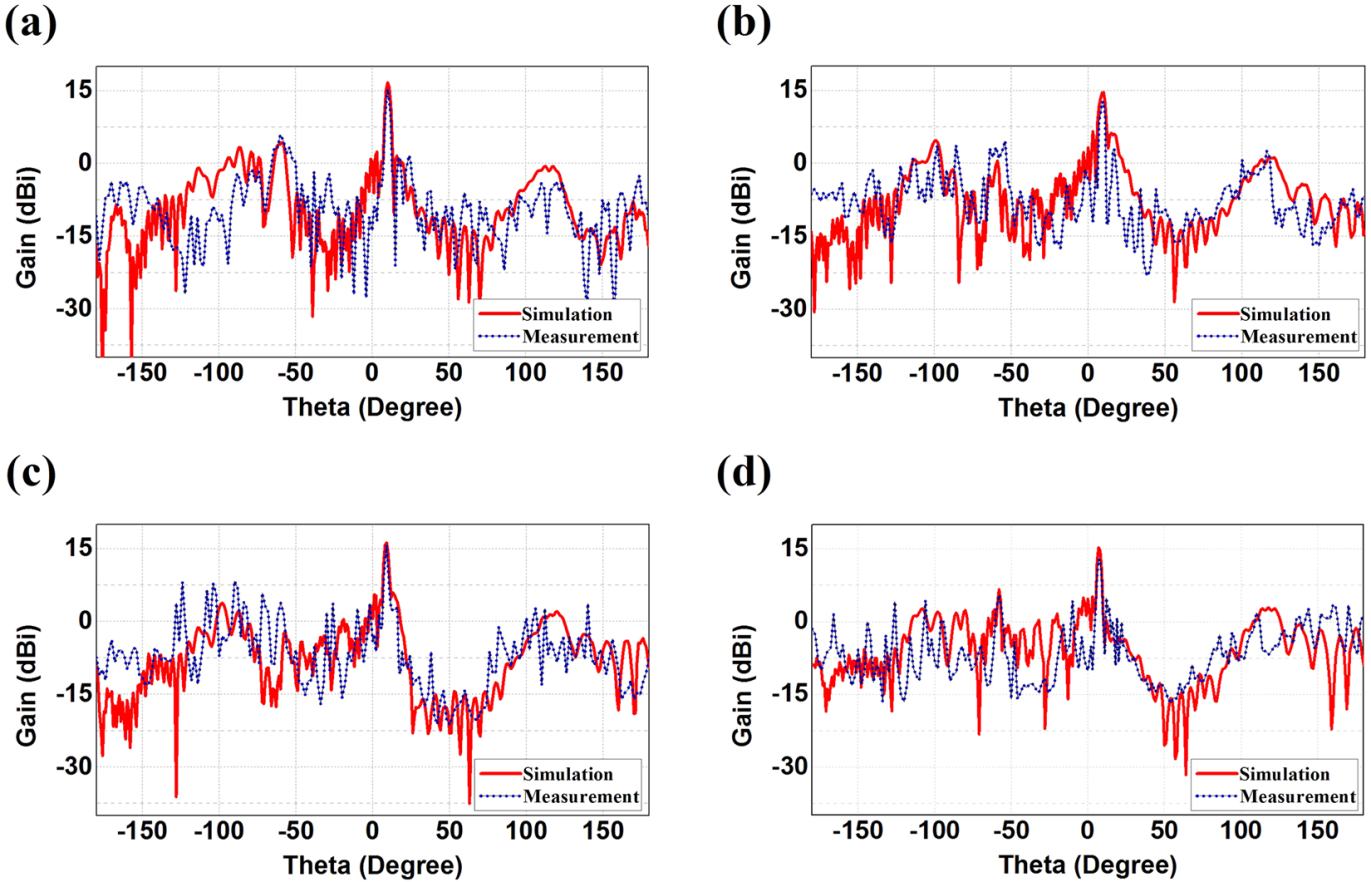
Comparisons of the measured radiation patterns and the simulation results of the dual reflecting system. (**a**) 15 GHz, (**b**) 15.3 GHz, (**c**) 15.5 GHz, and (**d**) 15.7 GHz.

Finally, we fabricate the proposed convex meta-mirror and the dual reflecting system and experimentally test the radiation performances, as demonstrated in Figs 7, 8 and 9. The meta-mirrors and the feeding source of WR62 waveguide are embedded in the parallel plate waveguide, where we can observe in Fig. 7 that both of the meta-mirrors are constructed on the aluminum bases which are coated with the ring patches on the FR4 grounded dielectric substrates. The aluminum bases here also function as the brackets to support the parallel plate waveguide. The ring patches are shown more clearly by the magnified pictures in the demonstration and dimensions of every unit cell of both meta-mirrors are manufactured according to the Table 1 and 2. We continue to demonstrate the radiation patterns of such a convex meta-mirror and the dual reflecting system in Figs 8 and 9 by comparing the measured results with the simulations. On the one hand, we can observe that the experimental plots and the simulation results of the convex meta-mirror match each other quite well over the examined frequency range, and the highly directive radiations from the convex meta-mirror are generated exactly as we expected. On the other hand, we can observe from the experimental tests of the dual reflecting system that all the far-field radiations from 15 GHz to 15.7 GHz present the highly directive radiations and the main beams are all steered at 10° as we devised for the negative reflection. We can also observe that the unwanted spectacular reflections from the experiments are a little higher than the simulations, and the ratios between the gains of negative reflections and the spectacular reflections are smaller than the simulation results. However, the overall performances shown from the experiments of the dual reflecting system still fulfill our initial design of the negative reflecting meta-mirrors, and further verify the functionalities of the dual reflecting system to obtain the plane wave from the convex meta-mirror firstly and then have the well collimated beam negative reflected by the planar meta-mirror.

In conclusion, we have proposed negative reflecting meta-mirrors in this paper and demonstrated that the incident wave can be reflected anomalously with a broad angle range of negative reflections. Applications for the planar meta-mirrors and the convex meta-mirrors are both demonstrated and practically implemented through a periodic arrangement of the subwavelength ring patches, where the impacts of the oblique incident angles have also been considered when generating the desired phase distributions. Finally, we carried out the experiments to verify the functionalities of the convex meta-mirror and the planar meta-mirror. Especially, we have further built up a dual reflector system with the demonstration of obtaining the plane wave from the convex meta-mirror and then having the well collimated beam negative reflected by the planar meta-mirror. We expect the proposed design of the negative reflecting meta-mirrors would be applicable to electromagnetic problems in relative research fields, especially for devising smart planar illusion devices, and highly directive antennas mounting on convex surfaces of various platforms.

## Electronic supplementary material

Supplementary Information
