# Supplementary material for: Negative Reflecting Meta-Mirrors
Source: Sci Rep. 2017 Jul 18;7:5729. doi: 10.1038/s41598-017-06184-1 (PMC5515955; doi:10.1038/s41598-017-06184-1)
Supplement: Supplementary file 1 — Supplementary Information [file 41598_2017_6184_MOESM1_ESM.doc]

**Negative Reflect****ing Meta-Mirrors**

Rui Yang^*^, Dong Li, Dongxing Gao, Aofang Zhang, Bowei Hu, Pei Yang, Zhenya Lei, and Jiacheng Li

School of Electronic Engineering. Xidian University, Xi'an 710071, People’s Republic of China

*[ruiyang.xidian@gmail.com](mailto:ruiyang.xidian@gmail.com)


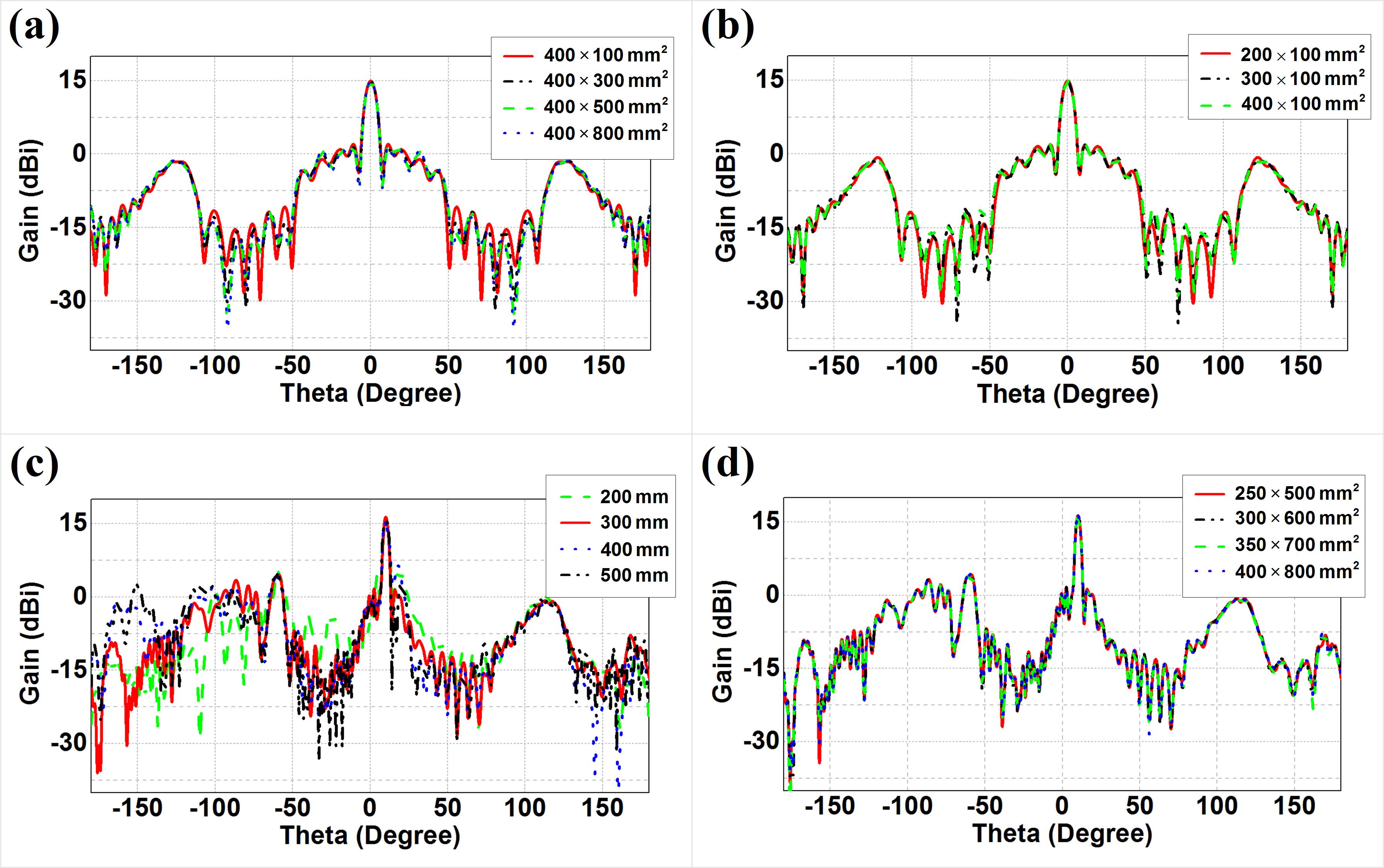


**Figure S1.**  Optimization of the convex meta-mirror and the dual reflecting system. (a) Radiation performance of the convex meta-mirror varied with the length of the parallel plate waveguide. (d) Radiation performance of the convex meta-mirror varied with the width of the parallel plate waveguide. (c) Radiation performance of the dual reflecting system varied with the distance between the two meta-mirrors. The parallel plate waveguide is set to be 400 × 800 mm^2^. (d) Radiation performance of the dual reflecting system varied with the size of the parallel plate waveguide. The distance between the two meta-mirrors is 300 mm.
